# The Metallochaperone Encoding Gene *hypA* Is Widely Distributed among Pathogenic *Aeromonas* spp. and Its Expression Is Increased under Acidic pH and within Macrophages

**DOI:** 10.3390/microorganisms7100415

**Published:** 2019-10-02

**Authors:** Ana Fernández-Bravo, Loida López-Fernández, Maria José Figueras

**Affiliations:** Unit of Microbiology, Department of Basic Health Sciences, Faculty of Medicine and Health Sciences, IISPV, University Rovira i Virgili, 43201 Reus, Spain; ana.fernandez@urv.cat

**Keywords:** metallochaperone, *Aeromonas*, macrophages, acid, alkaline, ROS, hydrogenases, ureases

## Abstract

Metallochaperones are essential proteins that insert metal ions or metal cofactors into specific enzymes, that after maturation will become metalloenzymes. One of the most studied metallochaperones is the nickel-binding protein HypA, involved in the maturation of nickel-dependent hydrogenases and ureases. HypA was previously described in the human pathogens *Escherichia coli* and *Helicobacter pylori* and was considered a key virulence factor in the latter. However, nothing is known about this metallochaperone in the species of the emerging pathogen genus *Aeromonas*. These bacteria are native inhabitants of aquatic environments, often associated with cases of diarrhea and wound infections. In this study, we performed an in silico study of the *hypA* gene on 36 *Aeromonas* species genomes, which showed the presence of the gene in 69.4% (25/36) of the *Aeromonas* genomes. The similarity of *Aeromonas* HypA proteins with the *H. pylori* orthologous protein ranged from 21−23%, while with that of *E. coli* it was 41−45%. However, despite this low percentage, *Aeromonas* HypA displays the conserved characteristic metal-binding domains found in the other pathogens. The transcriptional analysis enabled the determination of *hypA* expression levels under acidic and alkaline conditions and after macrophage phagocytosis. The transcriptional regulation of *hypA* was found to be pH-dependent, showing upregulation at acidic pH. A higher upregulation occurred after macrophage infection. This is the first study that provided evidence that the HypA metallochaperone in *Aeromonas* might play a role in acid tolerance and in the defense against macrophages.

## 1. Introduction

Metal ions are essential for the correct function of microbial biological processes [1]. In fact, many proteins contain metal ions bound directly to their amino acid chains by histidine or cysteine residues as cofactors. Particularly, metalloenzymes such as nitrogenases, ureases or hydrogenases are abundant types of metalloproteins that catalyze numerous metabolic and enzymatic reactions [2,3,4]. The synthesis of these metalloenzymes consists of complex processes that require a set of accessory proteins. In this context, metallochaperones play a key role in bacterial metal homeostasis (“metallostasis”) since they are involved in the acquisition and transfer of metals [4,5,6]. Many lines of evidence indicate an important role of metallostasis in the host-pathogen interaction [1,6,7]. For instance, during infection the host limits the availability of essential metals, inactivating metal-dependent processes of the bacterial pathogen, which compensates for this limitation by producing metallochaperones, among other proteins.

Metallochaperones act directly, inserting metal ions into specific enzymes that will become metalloenzymes after maturation [4,5,6]. One of the best-studied regulation mechanisms by metallochaperones in bacteria is the maturation of hydrogenases, which are enzymes that catalyze the interconversion of hydrogen (H_2_) into protons and electrons, playing a vital role in anaerobic metabolism and oxidative stress response [8]. There are three types of hydrogenase based on the metal attached to their active site: [FeFe] hydrogenase, [NiFe] hydrogenase and [Fe]-only hydrogenase [9,10]. The [NiFe]-hydrogenases are heterodimeric proteins consisting of two subunits, a small (~26 kDa) and a large (~62 kDa) which possesses in its active site the metallic cofactor NiFe(CN)_2_CO responsible for catalyzing the reversible production of molecular H_2_ [9]_._ Several accessory proteins encoded by genes present in the *hyp* operon are required for NiFe(CN)_2_CO biosynthesis [11,12]. In fact, the HypC, HypD, HypE and HypF proteins are considered hydrogenase maturation factors and are responsible for the synthesis and transfer of the Fe(CN)_2_ complex to the hydrogenase precursor. After delivery of the Fe(CN)_2_CO complex to the hydrogenase, the metallochaperone HypA and the GTPase HypB participate in its correct maturation by transferring a nickel ion into the large subunit of the hydrogenase [11,13].

The nickel-binding HypA metallochaperone has been well studied in the human pathogens *Helicobacter pylori* and *Escherichia coli,* in which it has been related to virulence [11,14,15,16,17]. Although the function of this metallochaperone is mainly associated with the maturation of hydrogenases, recent studies have demonstrated that HypA is also involved in urease maturation [17,18,19]. Both metalloenzymes are relevant in the adaptation to several redox conditions in pathogenic bacteria. Previous studies demonstrated that the hydrogenases participate in the defense against oxidative stress [20], as well as in acid resistance in *E. coli* [17,21,22]. The data showed that an *E. coli* K-12 hydrogenase mutant exhibits impaired acid resistance [17,21,22]. Moreover, previous works revealed that ureases facilitate the survival of *H. pylori* in the human gastric mucosa by protecting it from the acidic environment of the stomach [17,18,19]. The ureases neutralize the gastric acids by catalyzing the conversion of urea into ammonia and carbon dioxide. The latter studies demonstrated that the modification of the zinc- and nickel- binding sites in HypA affects its urease activity and consequently impairs acid resistance in this bacterium [17,18,19].

An earlier study investigated the redox potential of facultative aerobic and obligate anaerobic bacteria that produce H_2_ along the gut of earthworms by analyzing the [NiFe]- and [FeFe]-hydrogenase gene transcripts [23]. The results showed that 21% of the detected [NiFe]-hydrogenase-affiliated sequences corresponded to bacteria of the genus *Aeromonas* [23]. Therefore, these findings evidence the fermentative capacity of these bacteria as great hydrogen producers. Although the presence of hydrogenases has been described in *Aeromonas,* nothing is known about the function of the metallochaperone HypA in this genus [23]. *Aeromonas* species are considered opportunistic emergent pathogens producing mainly gastroenteritis and 96.5% of the clinical strains correspond to four species: *Aeromonas caviae* (29.9%), *Aeromonas dhakensis* (26.3%), *Aeromonas veronii* (24.8%) and *Aeromonas hydrophila* (15.5%). In addition, few *Aeromonas* species typically associated with high mortality infections in fish, such as *A. salmonicida,* have been reported to display strains able to produce human disease. [24,25,26]. Hence, it is of interest to investigate whether the HypA metallochaperone could be considered a new potential virulence factor of these bacteria. Therefore, the objective of this study was to search for the presence of the *hypA* gene in the genomes of the species of the genus *Aeromonas,* as well as to evaluate if HypA participates in their adaptive molecular response to acidic conditions and oxidative stress.

## 2. Materials and Methods

### 2.1. Bacterial Strains and Culture Conditions

*Aeromonas dhakensis* CECT 5744^T^, *Aeromonas caviae* CECT 838^T^, *Aeromonas veronii* CECT 4257^T^*, Aeromonas hydrophila* CECT 839^T^, *Aeromonas salmonicida* CECT 894^T^ and *Aeromonas piscicola* CECT 7443^T^ type strains were used in this study and stored at −80 °C in Tryptic Soy Broth (TSB; Difco™, Bordeaux, France) with 20% of glycerol. Bacteria were grown in Tryptone Soya Agar (TSA; Difco™, Bordeaux, France) at 30 °C for 24 h. Prior to infection, bacteria were grown at 37 °C in serum and antibiotic-free Dulbecco’s Modified Eagle’s Medium (DMEM; Biowest, Nuaillé, France) for 18 h.

For evaluation of urease activity, the bacteria were grown in Urea Agar Base media (with a yellow-orange color) at 37 °C for 24 h and the change of color of the slant indicated a positive reaction. A strain of *Salmonella* sp. was used as a negative control and one of *Proteus* sp. as a positive control. The strains used in this study belong to our laboratory collection (Microbiology unit, University Rovira I Virgili)

### 2.2. In Silico Search of hypA and ure Genes in the Aeromonas Genomes

For identification of *hypA* sequences in *Aeromonas* species, an initial text-based search was conducted on UniProtKB from Uniprot database (https://www.uniprot.org/uniprot/). As a result, the hypA deduced amino acid sequence from *Aeromonas hydrophila* CECT 839^T^ (A0KL76) was found. The corresponding nucleotide sequence of this strain (ABK37593.1) was used as query for BLAST search on 36 genomes of *Aeromonas* spp. type strains deposited in the NCBI database (https://blast.ncbi.nlm.nih.gov/Blast.cgi) to identify *hypA* orthologues sequences. To investigate whether the presence of *hypA* could be a species-specific characteristic, we extended our analysis to other available genomes of non-type strains (*n* = 110). These genomes were verified with the Average Nucleotide Identity (ANI) using the online tool OrthoANI or with the analysis of the *rpoD* gene [27,28]. For identification of urease genes in the *Aeromonas* genomes, another BLAST search was performed using as query the sequences of the genes: *ureA, ureB, ureI, ureE, ureF, ureG* and *ureH* extracted from the genome of *H. pylori* strain HPAG1 (annotated in the NCBI database).

### 2.3. Protein Sequence Analysis and 3D-Structure Prediction

To determine sequence conservation of *hypA* in *Aeromonas*, a comparison of HypA proteins among the 25 *Aeromonas* species and one of *E. coli* (strain E24377A) and *H. pylori* (strain HPAG1) was assessed by multiple alignments, using the CLUSTALW algorithm via MegAlign. Phylogenetic relationships among sequences were depicted in a phylogenetic tree constructed with MEGA6 using the Neighbor-joining and Maximum-likelihood algorithms. In addition, the prediction of 3D monomeric and dimeric HypA protein structure and the comparative analyses of this protein with those of *A. hydrophila* (CECT 839^T^) and *E. coli* (E24377A), was done using the Swiss-model online tool (https://swissmodel.expasy.org/).

### 2.4. Cell Line Culture, Infection and Induction Experiments

The cell line J744A.1 from mouse BALB/C monocyte macrophages was used for the infection experiments with the six *Aeromonas* type strains. The macrophages cells were maintained in adhesion in Dulbecco’s Modified Eagle’s Medium (DMEM; Biowest, Nuaillé, France) (pH = 8) supplemented with 10% fetal bovine serum (FBS; Biowest, Nuaillé, France) plus 1% penicillin-streptomycin solution (P/S; Biowest, Nuaillé, France) at 37 °C and 5% CO_2_. Prior to infection, cells were seeded in tissue culture plates (1 × 10^6^ cells/mL) containing serum-free DMEM without antibiotics (serum-starvation conditions) for 18 h. The macrophages J774A.1 were infected with the six *Aeromonas* type strains grown in serum-free DMEM without antibiotics at a multiplicity of infection (MOI) of 5. In addition, bacteria were seeded onto tissue culture plates in serum-free DMEM without antibiotics at alkaline pH (pH = 8) or acidic pH (pH = 4.5) adjusted with an HCl solution followed by filtration to remove any precipitate. Co-cultures were incubated at 37 °C and 5% CO_2_ up to 4 h for gene expression analyses.

### 2.5. RNA Extraction and Quantitative RT-PCR

Total RNA was isolated from logarithmic-phase *Aeromonas* cultures using TRIzol^®^ Reagent (Invitrogen, Carlsbad, CA, USA) as previously described [29]. RNA quality and integrity were confirmed spectrophotometrically using Nanodrop 2000, calculating the 260/280 and 260/230 ratios. The cDNA was transcribed from RNA using iScript cDNA Synthesis Kit (Bio-Rad Laboratories, Inc. Hercules, CA, USA) according to the manufacturer’s instruction. Quantitative Real-Time PCR was performed in duplicate using Real-Power SYBR^®^ green PCR Mastermix (Applied Biosystems^®^, Waltham, MA, USA) in 10 µL total PCR reaction mixture on a StepOnePlus™ Real-Time PCR System (Applied Biosystems). The thermal cycling conditions were: 94 °C for 5 min, followed by 45 cycles of 30 s at 94 °C, 30 s at 60 °C, 30 s at 72 °C, and finally, 20 s at 80 °C. The threshold cycle (C*t*) was automatically determined by the StepOne Software v2.0 (Applied Biosystems) to calculate the relative expression of the tested gene (*hypA)* using as reference the 16S rRNA housekeeping gene, as previously described [30]. Relative gene expression levels and fold change expression were estimated using 2^−ΔΔC*t*^ method [31]. The specific primer pairs for the PCR amplification of *hypA* and 16S rRNA were designed by using consensus nucleotide sequences and Oligo Primer Analysis Software v. 7 (Table 1). Experiments were performed in triplicate using three independently prepared bacterial growth cultures obtained on three different days.

### 2.6. Statistical Analysis

All experiments were performed in triplicates and significant differences were determined using Student’s two-tailed *t*-test calculated using GraphPad Prism 6.0 (GraphPad Software, CA, USA). *p* values ≤ 0.05 were considered statistically significant (*).

## 3. Results

### 3.1. Identification of hypA Gene in Aeromonas Species

The results of the in silico search using the *hypA* sequence (339 bp) of *A. hydrophila* CECT 839^T^ as template showed that hypA was present in 69% (25/36) of investigated genomes of type strains belonging to the *Aeromonas* species shown in Table 2. Extended analyses on 108 additional genomes from those 36 *Aeromonas* species available in NCBI database, allowed investigation of whether *hypA* is a strain or a species-specific character (Table S1). We detected *hypA* gene sequences in 83% (122/146) of all genomes analyzed. In most species analyzed, concordance between strains regarding the presence/absence of hypA in their genomes is observed. Although it is interesting to mention that *A. caviae*, *A. schubertii* and *A. media* showed a discrepancy, since some of the strains have *hypA* and others do not (Table S1).

### 3.2. Sequence Analyses of hypA Proteins Shows Specific Motifs Associated to Metal Binding

**Figure 1:**
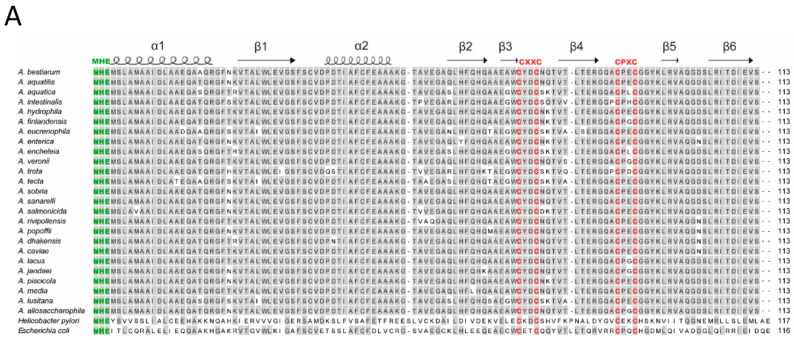

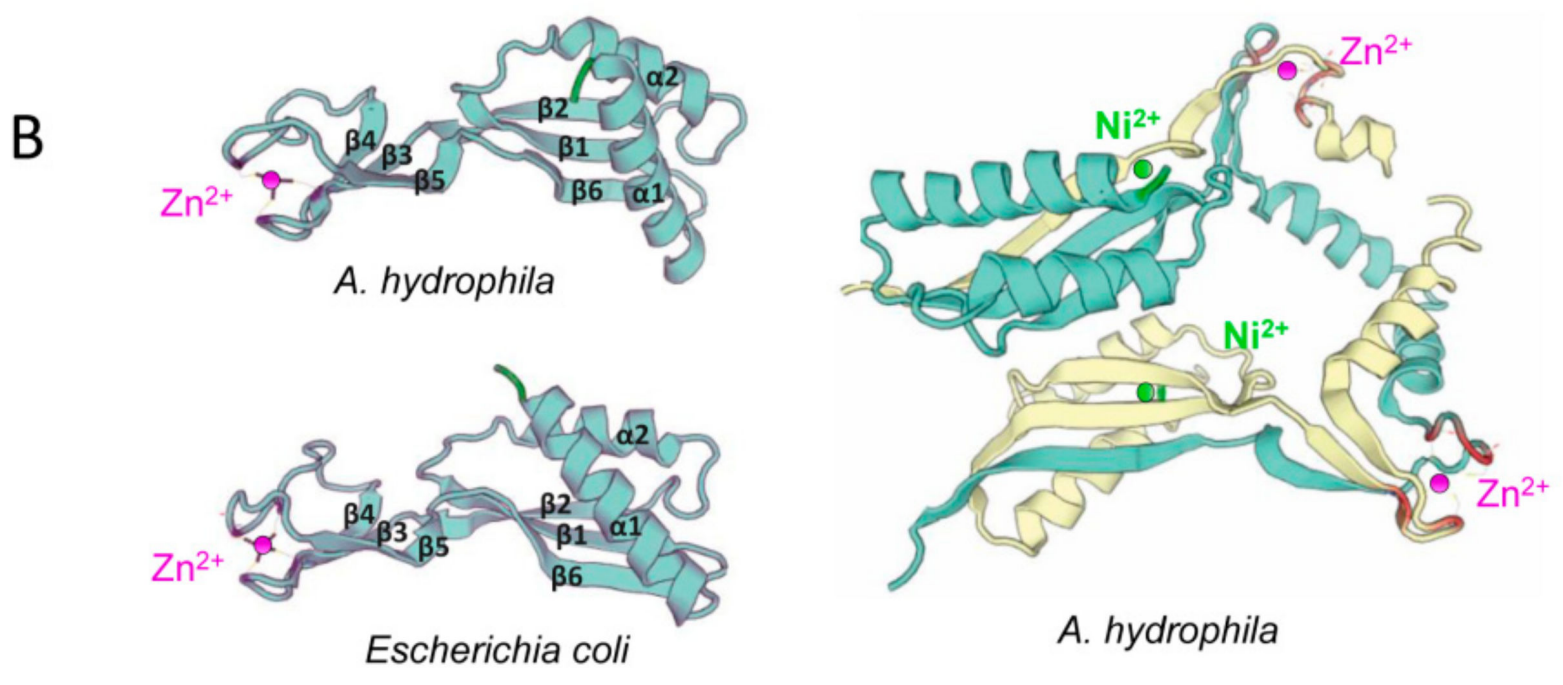
(**A**) Sequence alignment of the in silico-translated amino acid sequences of HypA proteins from 36 *Aeromonas* species, *E. coli* and *H. pylori*. The alignment was constructed with MegAlign. The MHE correspond to the motif of the Nickel binding domain (green) and CxxCnCPxP to the Zinc binding domain (red). The characteristic α-helices (wave lines) and a β-sheet (arrows) are represented. (**B**) Predicted monomeric and dimeric structure of HypA proteins from *A. hydrophila* type strain and *E. coli* constructed with Swiss Model online tool, the α-helices and the stranded β-sheet motifs are indicated.

The specific metal-binding motifs consisting of N-terminal MHE motif for Ni-binding and two consecutive cysteine motifs CxxCnCPxC for Zn-binding, previously reported in the HypA proteins of *E. coli* and *H. pylori*, were also observed in the *Aeromonas* spp. protein sequences in Figure 1A. The three-dimensional predicted structures of HypA proteins of *A. hydrophila* CECT 7996^T^ and *E. coli* show high similarity among them (Figure 1A). Indeed, monomeric and dimeric predicted proteins of the latter species displayed the characteristic α-helices (α1 and α2) and a β-sheet (long β1, β2, and β6 and short β3, β4, and β5) (Figure 1B).

### 3.3. Conservation and Phylogenetic Relationships of hypA in Aeromonas

**Figure 2:**
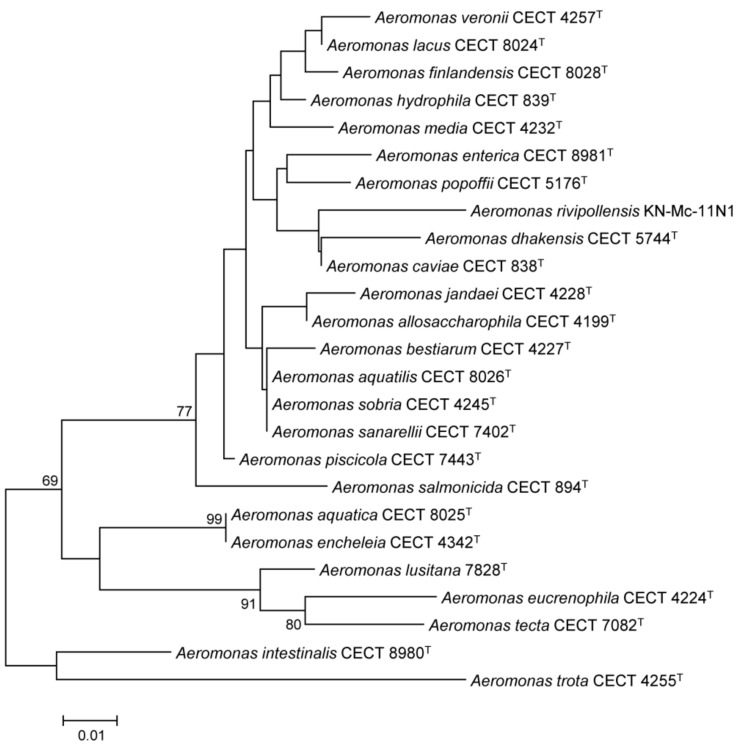
Phylogenetic tree constructed with the in silico-translated amino acid sequences of HypA proteins (113 aa) from 36 *Aeromonas* species type strains (the only sequence not belonging to the type strain corresponds to *A. rivipollensis* KN-Mc-11N1). The phylogenetic analysis was constructed with MEGA6, using Neighbor-joining algorithm. Numbers at nodes represent bootstrap percentages (>50%) obtained by repeating analysis 1000 times.

In silico HypA is the similarity of the in silico-translated amino acid sequences of HypA between the *Aeromonas* species ranged between 86% and 100%. As expected, when comparing the *Aeromonas* HypA with those from *E. coli* and *H. pylori* the similarity was significantly lower, 41–45% and 21–26% respectively (Figure 1A). As observed in the phylogenetic tree, HypA proteins were highly conserved among the 25 *Aeromonas* species. Two groups of species, one formed by *A. aquatilis*, *A. sobria* and *A. saranellii* and the other with the species *A. encheleia* and *A. aquatica* had identical hypothetical protein sequence (Figure 2 and Figure S1). Thus, in conclusion, we can state that HypA was highly conserved within the genus *Aeromonas.*

In addition, it is important to mention that there are many identified hypA encoded proteins across different genus and bacterial species, which provide evidence of their conservation throughout evolution (Table S2). However, little is known about the biological function of HypA proteins in the vast majority of these species.

### 3.4. Transcriptional Regulation of hypA under Different pH-Condition and Macrophage

**Figure 3:**
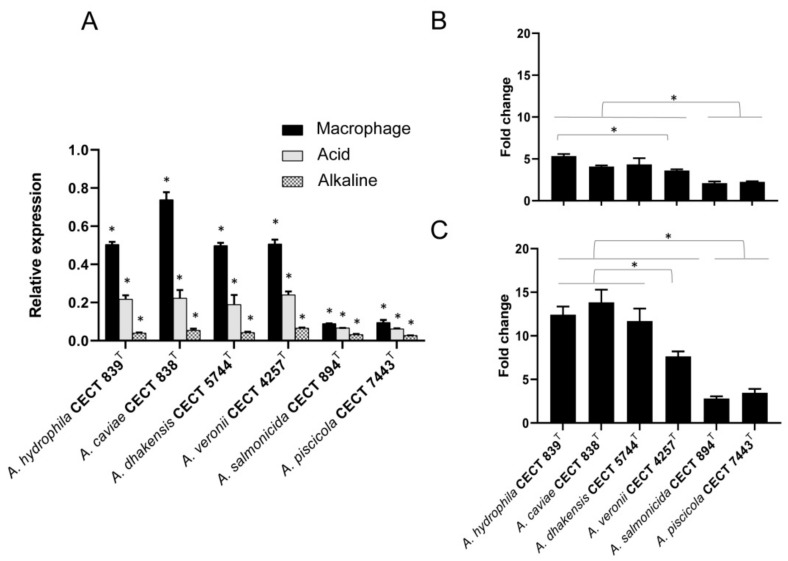
Expression of *hypA* in *Aeromonas* species determined by RT-qPCR. (**A**) Relative expression of *hypA* during phagocytosis by macrophages and under acidic and alkaline culture conditions. Transcript levels of *hypA* were normalized to the expression of 16S rRNA. (**B**) Expression fold change of *hypA* on bacteria grown on acid media respect to the cultured in alkaline media and (**C**) expression fold change on bacteria phagocytized by macrophages after 4 h of infection respect to control condition on culture alkaline media, calculated using the comparative ΔΔ*C*t method. Error bars indicate standard deviations calculated from three independent experiments. * *p*-value < 0.05.

The expression patterns of *hypA* determined under stressful pH conditions in the most prevalent clinical species (*A. hydrophila*, *A. caviae*, *A. dhakensis* and *A. veroni*) and in two species frequently associated with fish diseases (*A. salmonicida* and *A. piscicola*) by qRT-PCR are shown in Figure 3A. All *Aeromonas* species displayed a similar relative expression of *hypA* in alkaline conditions (pH 8) (Figure 3A). Nevertheless, the expression of *hypA* was significantly higher (*p* < 0.05) under acid condition (pH 4) in comparison to alkaline condition (pH 8), displaying a greater upregulation in the most prevalent clinical species (Figure 3A,B). Furthermore, given that the phagosome of macrophages becomes acid upon phagocytosis of pathogens, we evaluated the expression of *hypA* during *Aeromonas* infection. The results showed that *Aeromonas* upregulates *hypA* in response to phagocytosis, displaying a significantly higher expression of the metallochaperone during infection than in control (alkaline media) or in vitro acid exposure (*p* < 0.05) (Figure 3A,C). Although transcriptional regulation of *hypA* seems to depend on pH or infection condition, the statistical analysis revealed strain-related differences. The most clinically prevalent species showed a significantly higher upregulation of *hypA* under acid exposure and during infection than species considered environmental or more related to fish disease (Figure 3B,C). Significant differences in gene induction under acid exposure were observed among the most prevalent clinical species, except between *A. hydrophila* and *A. veronii* (Figure 3B). Additionally, there were significant differences in the group of clinically prevalent species, with *A. veronii* showing notably lower induction of *hypA* when compared with *A. hydrophila*, *A. caviae* and *A. dhakensis* during macrophage infection (*p* < 0.05) (Figure 3C).

**Figure 4:**
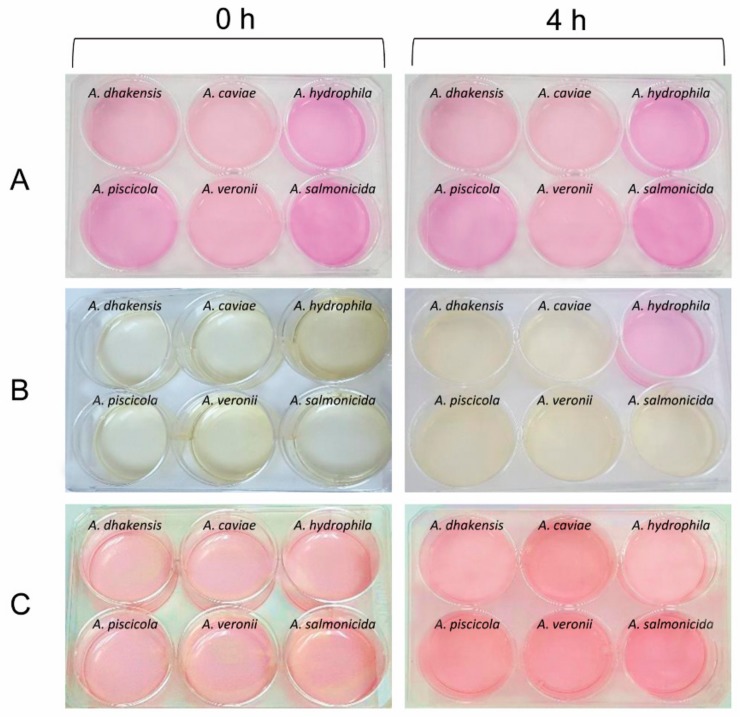
Culture plates with different *Aeromonas* species at 0 and 4 h. (**A**) Bacteria culture in alkaline medium DMEM. (**B**) Bacteria culture in acid DMEM with HCl. (**C**) Macrophages in alkaline DMEM infected by different *Aeromonas*. Yellow indicates acid pH and pink indicates alkaline pH.

With the purpose of understanding how *Aeromonas* adapts to low pH environments, we determined the pH variations of the medium during in vitro growth or during infection of macrophages with the species under study. No significant changes in pH were observed when strains where grown in DMEM at pH 4 or pH 8 with the only exception of *A. hydrophila* (CECT 839^T^). When the latter strain was incubated in DMEM at pH 4 there was an increase of pH that reached up to 7.5 and that was visually evident by the changing color of the DMEM media, that functions as a pH indicator (Figure 4A,C). This basification of the medium or other pH changes were not observed for other species, neither for the infected or uninfected macrophages (Figure 4C).

### 3.5. Urease Activity and Urease Genes in Aeromonas Species

HypA is also involved in the urease maturation which facilitates the survival of bacteria in the human gastric mucosa neutralizing the acidic environment [17]. Therefore, we have evaluated the ability of *Aeromonas* to hydrolase urea by determining urease activity using a biochemical method. None of the strains assayed produced ureases because no color change in the media from light orange to magenta was observed when compared with the positive urease control (*Proteus* sp.). In addition, the battery of proteins associated with urease activity was not found in the genome of the six strains under study [32]. This is consistent with the absence of the urease genes in *Aeromonas* sp., which is inferred from our in silico search of all available genomes of the *Aeromonas* genus.

## 4. Discussion

The relevance of bacterial metal homeostasis is related to the essential role that metals play for survival in different environments, including the context of host-pathogen interaction during the infection processes [33]. In the last years, there has been significant progress in the knowledge of how metallochaperones bind metal ions, recognize the target proteins and facilitate metal transfer [4,6]. The HypA metallochaperone has been associated with [NiFe] hydrogenase and urease maturation and is considered a relevant protein for adaptation to acidic environments of pathogenic bacteria like *H. pylori* and *E. coli* [17,18,19]. Furthermore, the HypA metallochaperone participates in the defense against oxidative environments [20].

The present work is the first to address the study of the HypA metallochaperone in the genus *Aeromonas.* Our results suggest that *hypA* genes in certain species, like *A. hydrophila, A. dhakensis*, *A. veronii* and *A. taiwanensis* among others, are widely conserved among their strains. However, other species like *A. caviae*, *A. schubertii* and *A. media* showed strain-level variants, in which some strains from the same species contain *hypA* and others do not. Such lack of uniformity in *hypA* presence in well-represented species, as *A. veroni*, would indicate within-species microevolutionary changes that could result from environmental adaptation [34]. On the other hand, in our study some species are poorly represented, and additional analyses should be performed in order to give precise conclusions. Overall these data indicate that *hypA* tends to be conserved within species although cannot be considered strictly a species-specific character.

The *Aeromonas* HypA protein sequences showed to be moderately similar to those described in *E. coli* and *H. pylori.* However, they display the characteristic *N*-terminal MHE motif for Ni-binding and two consecutive cysteine motifs CXXCnCPXC for Zn-binding, also conserved in the tow human pathogenic bacteria mentioned [13,14,16,17]. In addition, the three-dimensional structure predicted for all proteins showed to be highly similar among the *Aeromonas* species and the other two human pathogenic bacteria, confirming that it is a metallochaperone [15,16,35].

Although the principal role of HypA has been typically associated with the maturation of hydrogenases, in the last years it has been demonstrated that HypA could play a role in the maturation of ureases. These metalloenzymes are involved in the survival of pathogenic bacteria in an acidic environment. For instance, bacterial ureases are involved in the survival of *H. pylori* in the human stomach at acidic pH [17,18,19]. In *E. coli* this function is carried out by other enzymes i.e., hydrogenases [17,21,22]. The fact that 80% of infections caused by *Aeromonas* are gastrointestinal diseases indicates an adaptation of these bacteria to acid environments of the gastrointestinal tract [25,26]. Additionally, a previous study hypothesized that urease activity may contribute to acid tolerance in some *A. caviae* strains, facilitating bacterial survival during infection, as occurs in *Yersinia enterocolitica* [36]. However, *Aeromonas* species usually have been described as urease negative [37]. Consistent with these previous data we observed in our study that all strains were urease negative. However, the expression study demonstrates that *hypA* is upregulated in acidic pH. A reasonable explanation could be that hydrogenases, but not ureases, would be involved in acid resistance in *Aeromonas*, as occurs in *E. coli* [18,22,23]. In addition, our results showed that *A. hydrophila* CECT 839^T^ alkalinize the medium during acid exposure. One feasible explanation for this phenomenon would be that *A. hydrophila* has a higher tolerance to acids as a consequence of an enzymatic pH shifting, which allows a better survival. Although additional analysis including more strains should be performed to determine if this is a specific characteristic of this species. Therefore, it would seem plausible to affirm that HypA could be associated with acid tolerance in *Aeromonas* species.

Numerous studies provide insights into the relevance of redox signaling and reactive oxygen species (ROS) production as defense mechanisms against pathogens [38,39]. Moreover, bacterial infections also can induce oxidative stress, which contributes to increase the rates of DNA mutations in the host. For instance, *H. pylori* produce superoxide anion in order to counteract the toxic effect of the ROS produced in the human stomach, which contributes even more in the development of gastric cancer [40]. Considering that the immune system generates ROS as defense mechanism against pathogens after phagocytosis by macrophages [41,42], resistance to acidic environments can be of great advantage for pathogens. Indeed, some studies have emphasized a strong relationship between deficiency of ROS production and susceptibility to microbial infection [43]. In these contexts, the discovery of the chaperone Hsp33 and its role in protecting cells against the deleterious effects of reactive oxygen species [44,45,46], reinforces the hypothesis of the important role of redox regulation during bacterial colonization [47]. In our study, the results showed that the *Aeromonas* metallochaperone HypA was upregulated after phagocytosis of macrophages, which is in line with the previous works [44,45,46]. Therefore, considering that hydrogenases participate in oxidative stress defense [20] it is very possible that HypA also contributes to the defense against ROS produced by macrophages in the phagocytic process.

According to the literature, 96.5% of the clinical *Aeromonas* strains correspond to four species: *Aeromonas caviae* (29.9%), *Aeromonas dhakensis* (26.3%), *Aeromonas veronii* (24.8%) and *Aeromonas hydrophila* (15.5%). However, other species usually associated with a fish disease like *A. salmonicida* have been isolated from human infections [25]. Our results showed a higher upregulation of the *hypA* after infection with the most prevalent clinical species, independently of the condition. Specifically, one strain used in the study (*A. hydrophila* CECT 839^T^) was isolated from milk although its virulence was previously demonstrated [48]. Otherwise, the expression of the *hypA* after infection with the strain *A. salmonicida* CECT 894^T^, described as a pathogenic for fish, was lower. These results could be associated with that possibility of a more important role of *hypA* in human health compared to animals, as is the case with other human pathogens as *E. coli* and *H. pylori.* However, further studies with a higher number of strains to better corroborate this hypothesis are needed.

In conclusion, our results suggest that HypA could play a role in the survival of *Aeromonas* in acidic environments and in defense against macrophages, although the exact mechanism remains unclear, but the possible role of this metallochaperone in *Aeromonas* sp. virulence is evident.

## 5. Conclusions

This study reports for the first time the distribution of orthologous sequences coding for the metallochaperone HypA in bacterial genomes of the genus *Aeromonas* and their deduced protein structure. Interestingly, HypA was present in 69.4% of *Aeromonas* species showing high similarity among the species (%). Metallochaperones are relevant proteins in the host-pathogen interaction. Thus, the present study suggests a possible role of HypA in bacterial survival in acidic environments, as well as in the defense against ROS produced by macrophages. In addition, it may promote future studies to confirm and better understand the function of this metallochaperone in the survival of species from the genus *Aeromonas*.

## Figures and Tables

**Table 1:** Oligonucleotides used in this study for transcriptional analysis.

Primers		Sequence 5′−3′
*hypA*	Forward	ATGCACGAAATGTCTCTGGC
	Reverse	TCGTAATTTGTACCCGCCAC
*16S rRNA*	Forward	TGTGTCCTTGAGACGTGGC
	Reverse	ACAAAGGACAGGGGTTGCG

**Table 2:** Presence/absence of *hypA* gene in the genomes of 36 *Aeromonas* species described until now.

Species	Strain	Source	Presence of *hypA* Gene	Protein Accession Number (NCBI)
*A. aquatica*	CECT 8025^T^	Cyanobacterial bloom	Yes	WP_033130233.1 ^¥^
*A. aquatilis* ^π^	CECT 8026^T^	Lake water	Yes	-
*A. allosaccharophila*	CECT 4199^T^	Eel	Yes	WP_042658210.1
*A. bestiarum*	CECT 4227^T^	Sick fish	Yes	WP_043555398.1
*A. caviae*	CECT 838^T^	Guinea pig	Yes	WP_017786826.1
*A. dhakensis*	CECT 5744^T^	Children feces	Yes	WP_042008198.1
*A. eucrenophila*	CECT 4224^T^	Fresh water fish	Yes	WP_042642402.1
*A. encheleia*	CECT 4342^T^	Eel	Yes	WP_033130233.1 ^¥^
*A. enterica* ^π^	CECT 8981^T^	Human feces	Yes	-
*A. finlandensis*	CECT 8028^T^	Cyanobacterial bloom	Yes	WP_033136932.1
*A. intestinalis* ^π^	CECT 8980^T^	Human feces	Yes	-
*A. jandaei*	CECT 4228^T^	Human feces	Yes	WP_042029441.1
*A. hydrophila*	CECT 839^T^	Milk	Yes	WP_005333346.1
*A. lacus*	CECT 8024^T^	Cyanobacterial bloom	Yes	-
*A. lusitana*	CECT 7828^T^	Untreated water	Yes	WP_100861683.1
*A. media*	CECT 4232^T^	Fisheries water	Yes	WP_042061779.1
*A. popoffi*	CECT 5176^T^	Drinking water	Yes	WP_042034298.1
*A. piscicola*	CECT 7443^T^	Sick fish	Yes	WP_021140355.1
*A. rivipollensis* *	KN-Mc-11N1	Wild nutria	Yes	WP_017778896.1
*A. salmonicida*	CECT 894^T^	Salmon	Yes	WP_005315136.1
*A. sanarelli*	CECT 7402^T^	Wound infection	Yes	WP_005301911.1 ^#^
*A. sobria*	CECT 4245^T^	Fish	Yes	WP_005301911.1 ^#^
*A. tecta*	CECT 7082^T^	Children feces	Yes	WP_050720085.1
*A. trota*	CECT 4255^T^	Human feces	Yes	WP_026458218.1
*A. veronii*	CECT 4257^T^	Sputum	Yes	WP_005351492.1
*A. australiensis*	CECT 8023^T^	Irrigation water	No	-
*A. bivalvium*	CECT 7113^T^	Shellfish	No	-
*A. cavernicola*	CECT 7862^T^	Cavern creek water	No	-
*A. crassostreae* ^π^	CECT 8982^T^	Shellfish	No	-
*A. diversa*	CECT 4254^T^	Wound infection	No	-
*A. fluvialis*	CECT 7401^T^	River water	No	-
*A. molluscorum*	CECT 5864^T^	Shellfish	No	-
*A. rivuli*	CECT 7518^T^	River water	No	-
*A. schubertii*	CECT 4240^T^	Skin abscess	No	-
*A. simiae*	IBS S-6874^T^	Monkey feces	No	-
*A. taiwanensis*	CECT 7403^T^	Wound infection	No	-

^T^ Type strain. * The genome of this strain was used because the genome of the type strain is not available. ^#,¥^ Same protein accession number because their gene product display 100% sequence identity. *^π^* Species pending to be described and draft genomes not freely available.

## Supplementary Materials

The following are available online at https://www.mdpi.com/2076-2607/7/10/415/s1.
