# Supplementary material for: The Metallochaperone Encoding Gene hypA Is Widely Distributed among Pathogenic Aeromonas spp. and Its Expression Is Increased under Acidic pH and within Macrophages
Source: Microorganisms. 2019 Oct 2;7(10):415. doi: 10.3390/microorganisms7100415 (PMC6843854; doi:10.3390/microorganisms7100415)
Supplement: Supplementary file 1 [file microorganisms-07-00415-s001.pdf]

Supplementary material

The Metallochaperone Encoding Gene hypA Is Widely Distributed among Pathogenic Aeromonas spp. and Its Expression Is Increased under Acidic pH and within Macrophages.

Ana Fernández-Bravo, Loida López-Fernández*, Maria José Figueras*.

Unit of Microbiology, Department of Basic Health Sciences, Faculty of Medicine and Health Sciences, IISPV, University Rovira i Virgili, Reus, Spain.

*Correspondence: Dr. Maria José Figueras, mariajose.figueras@urv.cat; Dr. Loida López-Fernández, loida.lopez@urv.cat

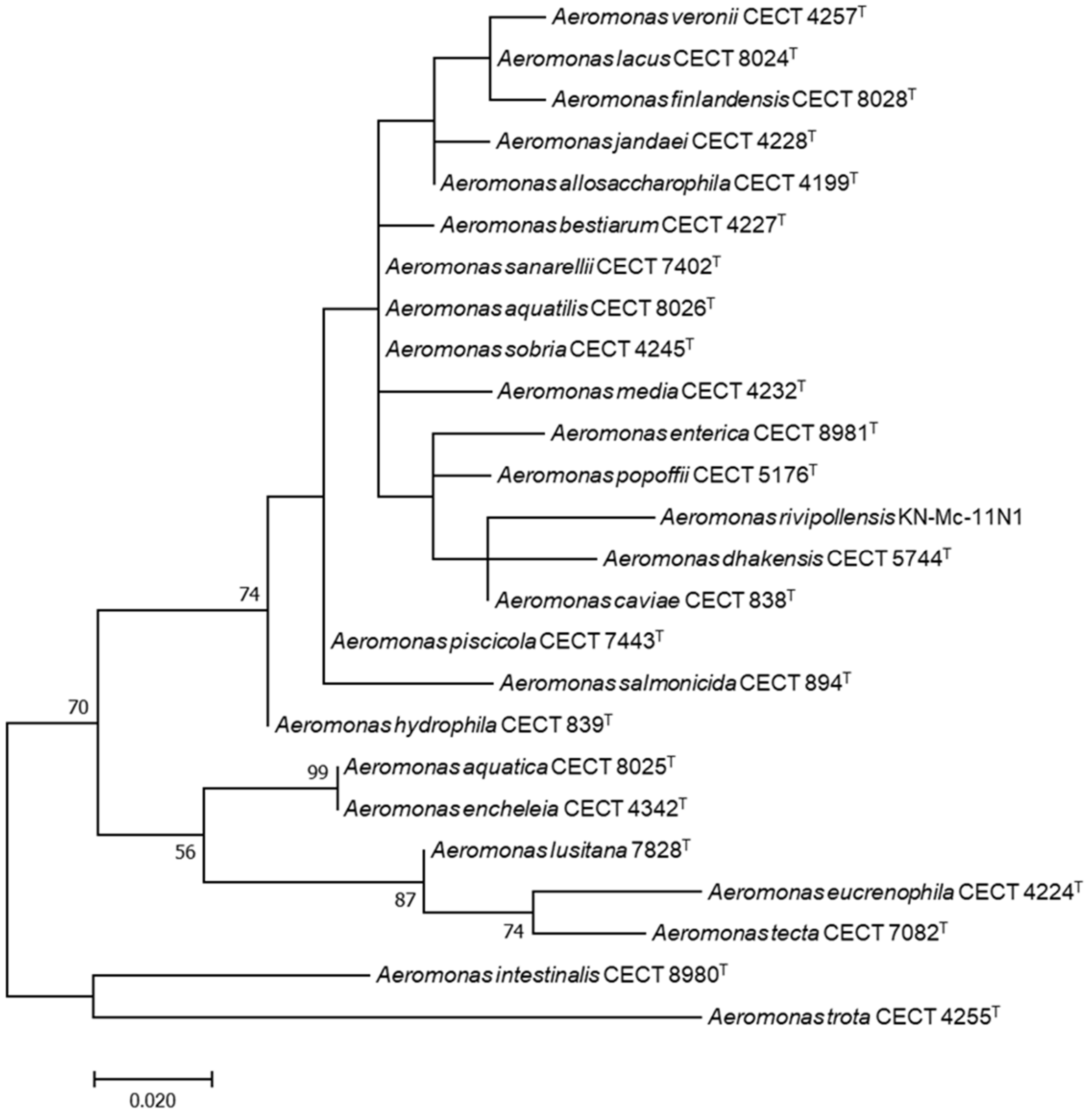

Figure S1. Phylogenetic tree constructed with 25 species of *Aeromonas* based on sequences of the protein HypA by the Maximum-Likelihood algorithm (model JTT+G). Numbers at nodes denote the level of bootstrap based on 1000 replicates; only values greater than 50% are shown. Scale bar, base substitutions per site.

Table S1. Genetic analyses and diversity of *hypA* presence at strain-level in 143 genomes from 36 different species from the genus *Aeromonas*. ^{T} indicates type strain.

Species	Strain	Identified (NCBI)	Accesion number (genome)	Re-identification	HypA Presence	Rate of hypA presence (%)
A. allosaccharophila	CECT 4199 ^{T}	*A. allosaccharophila*	NZ_CDBR00000000.1	-	Yes	4/4 (100)
	BVH88	*A. allosaccharophila*	NZ_CDCB00000000.1	*A. allosaccharophila*	Yes	
	Z9-6	*A. allosaccharophila*	NZ_NXBS00000000.1	*A. allosaccharophila*	Yes	
	TTU2014-	*A. allosaccharophila*	NZ_CDCB00000000.1	*A. allosaccharophila*	Yes	
A. bestiarum	CECT 4227 ^{T}	*A. bestiarum*	NZ_CDDA00000000.1	-	Yes	2/2 100
	CA23	*Aeromonas sp.*	NZ_CP023818.1	*A. bestiarum*	Yes	
A. bivalvium	CECT 7113 ^{T}	*A. bivalvium*	NZ_CDBT00000000.1	-	No	3/3 100
	ZJ19-2	*A.bivalvium*	NZ_NXBQ00000000.1	*A.bivalvium*	No	
	ZJ20-2	*A.bivalvium*	NZ_NXBX00000000.1	*A.bivalvium*	No	
A. caviae	CECT 838 ^{T}	*A. caviae*	NZ_CDBK00000000.1	-	Yes	14/20 (70)
	FDAARGOS_7	*A. caviae*	NZ_CP026055.1	*A. caviae*	Yes	
	NCTC12244	*A. caviae*	NZ_LS483441.1	*A. caviae*	Yes	
	R25-6	*A. caviae*	NZ_CP025705.1	*A. caviae*	Yes	
	ASNIH3	*Aeromonas sp.*	NZ_CP026222.1	*A. caviae*	Yes	
	8LM	*A. caviae*	NZ_CP024198.1	*A. caviae*	Yes	
	Aer593	*A. caviae*	NZ_RQJZ00000000.1	*A. caviae*	Yes	
	CHZ306	*A. caviae*	NZ_MDSC00000000.1	*A. caviae*	Yes	
	HAMBI_1972	*A. caviae*	NZ_QLLA00000000.1	*A. caviae*	Yes	
	FDAARGOS_7	*A. caviae*	NZ_JTBH00000000.2	*A. caviae*	Yes	
	GEO_48_Eff_A	*A. caviae*	NZ_QKOS00000000.1	*A. caviae*	Yes	
	GEO_47_Up_A	*A. caviae*	NZ_QKOU00000000.1	*A. caviae*	Yes	
	CH129	*A. caviae*	NZ_MDSD00000000.1	*A. caviae*	Yes	
	S2-003-000-	*A. media*	QFQK00000000.1	*A. caviae*	Yes	
	T25-39	*A. caviae*	NZ_CP025706	*A. caviae*	No	
	WCW1-2	*A. caviae*	NZ_CP039832	*A. caviae*	No	
	GSH8M-1	*A. caviae*	NZ_AP019195	*A. caviae*	No	
	R25-2	*A. caviae*	NZ_CP025777	*A. caviae*	No	
	ASNHI7	*Aeromonas sp.*	NZ_CP026226	*A. caviae*	No	
	ASNHI1	*Aeromonas sp.*	NZ_CP026228	*A. caviae*	No	
A. dhakensis	CECT 5744 ^{T}	*A. dhakensis*	NZ_CDBH00000000.1	-	Yes	10/10 (100)
	BVH70	*A. dhakensis*	NZ_NKWO00000000.1	*A. dhakensis*	Yes	
	BVH68	*A. dhakensis*	NZ_NKWQ00000000.1	*A. dhakensis*	Yes	
	BVH69	*A. dhakensis*	NZ_NKWP00000000.1	*A. dhakensis*	Yes	
	KN-Mc-6U21	*A. dhakensis*	NZ_CP023141.1	*A. dhakensis*	Yes	
	Cr2	*A. dhakensis*	NZ_LBDJ00000000.1	*A. dhakensis*	Yes	
	Aer283	*A. dhakensis*	NZ_RQKD00000000.1	*A. dhakensis*	Yes	
	277	*A. dhakensis*	NZ_AOBQ00000000.1	*A. dhakensis*	Yes	
	KOR1	*A. dhakensis*	NZ_LJOE00000000.1	*A. dhakensis*	Yes	
	L14h	*A. jandaei*	NZ_JWJR00000000.1	*A. dhakensis*	Yes	
A. hydrophila	CECT 839 ^{T}	*A. hydrophila*	NC_008570.1	-	Yes	33/33 (100)
	Ae34	*A. hydrophila*	NZ_BAXY00000000.1	*A. hydrophila*	Yes	
	AH10	*A. hydrophila*	NZ_CP011100.1	*A. hydrophila*	Yes	
	AL06-01	*A. hydrophila*	SAMN01085623	*A. hydrophila*	Yes	
	AL10-121	*A. hydrophila*	NZ_LRRW00000000.1	*A. hydrophila*	Yes	
	226	*A. hydrophila*	NZ_JEML00000000.1	*A. hydrophila*	Yes	
	ML09-121	*A. hydrophila*	NZ_LRRX00000000.1	*A. hydrophila*	Yes	
	AD9	*A. hydrophila*	NZ_JFJO00000000.1	*A. hydrophila*	Yes	
	RB-AH	*A. hydrophila*	NZ_JPEH00000000.1	*A. hydrophila*	Yes	
	YL17	*A. hydrophila*	NZ_CP007518.2	*A. hydrophila*	Yes	

Species	Strain	Identified (NCBI)	Accesion number (genome)	Re-identification	HypA Presence	Rate of hypA presence (%)
	AL06-06	*A. hydrophila*	NZ_CP010947.1	*A. hydrophila*	Yes	
	KN-Mc-1R2	*A. hydrophila*	NZ_CP027804.1	*A. hydrophila*	Yes	
	ASNIH4	*Aeromonas sp.*	NZ_CP026217.1	*A. hydrophila*	Yes	
	GYK1	*A. hydrophila*	NZ_CP016392.1	*A. hydrophila*	Yes	
	D4	*A. hydrophila*	NZ_CP013965.1	*A. hydrophila*	Yes	
	JBN2301	*A. hydrophila*	NZ_CP013178.1	*A. hydrophila*	Yes	
	NJ-35	*A. hydrophila*	NZ_CP006870.1	*A. hydrophila*	Yes	
	J-1	*A. hydrophila*	NZ_CP006883.1	*A. hydrophila*	Yes	
	pc104A	*A. hydrophila*	NZ_CP007576.1	*A. hydrophila*	Yes	
	AL09-71	*A. hydrophila*	NZ_CP007566.1	*A. hydrophila*	Yes	
	WCHAH04509	*A. hydrophila*	NZ_CP028568.2	*A. hydrophila*	Yes	
	ZYAH72	*A. hydrophila*	NZ_CP016989.1	*A. hydrophila*	Yes	
	AHNIH1	*A. hydrophila*	NZ_CP016380.1	*A. hydrophila*	Yes	
	MS-18-37	*A. hydrophila*	NZ_CP033604.1	*A. hydrophila*	Yes	
	ZYAH75	*A. hydrophila*	NZ_CP016990.1	*A. hydrophila*	Yes	
	FC951	*A. hydrophila*	NZ_CP032839.1	*A. hydrophila*	Yes	
	MX16A	*A. hydrophila*	NZ_CP018201.1	*A. hydrophila*	Yes	
	WCX23[a]	*A. hydrophila*	NZ_CP038463.1	*A. hydrophila*	Yes	
	WCX23[a]	*A. hydrophila*	CP028418.1	*A. hydrophila.*	Yes	
	GSH8-2 DNA	*A. hydrophila*	NZ_AP019193.1	*A. hydrophila*	Yes	
	23-C-23	*A. hydrophila*	NZ_CP038465.1	*A. hydrophila*	Yes	
	4AK4	*A. hydrophila*	NZ_CP006579.1	*A. hydrophila*	Yes	
	AH10	*A. hydrophila*	NZ_CP011100.1	*A. hydrophila*	Yes	
A. jandaei	CECT 4228 [T]	*A. jandaei*	NZ_JWJR00000000.1	-	Yes	5/5 (100)
	Riv2	*A. jandaei*	NZ_JFDL00000000.1	*A. jandaei*	Yes	
	Aer337	*A. jandaei*	NZ_RQKB00000000.1	*A. jandaei*	Yes	
	IMET J	*A. jandaei*	NZ_PRCV00000000.1	*A. jandaei*	Yes	
	Ho603	*A. jandaei*	NZ_NKWG00000000.1	*A. jandaei*	Yes	
A. media	CECT 4232 [T]	*A. media*	NZ_CDBZ00000000.1	-	yes	3/4 (75)
	ARB13	*A. media*	NZ_JRBG00000000.1	*A. media*	Yes	
	ARB20	*A. media*	NZ_JRBG00000000.1	*A. media*	Yes	
	WS	*A. media*	NZ_LR134376.1	*A. media*	No	
A. rivipollensis	KN-Mc-11N1	*A. rivipollensis*	NZ_CP027856.1	-	Yes	3/3 (100)
	BAQ071013-132	*A. media*	NZ_NKWW00000000.1	*A. rivipollensis*	Yes	
	Z1-6	*A. media*	NZ_NXBV00000000.1	*A. rivipollensis*	Yes	
A. salmonicida	CECT 894 [T]	*A. salmonicida*	NC_009348.1	-	yes	10/10 (100)
	O23A	*A. salmonicida*	NZ_CP021654.1	*A. salmonicida*	Yes	
	34mel	*A. salmonicida*	NZ_CP022426.1	*A. salmonicida*	Yes	
	01-B526	*A. salmonicida*	NZ_CP027000.1	*A. salmonicida*	Yes	
	RFAS1	*A. salmonicida*	NZ_CP017143.1	*A. salmonicida*	Yes	
	S68	*A. salmonicida*	NZ_CP022186.1	*A. salmonicida*	Yes	
	S44	*A. salmonicida*	NZ_CP022181.1	*A. salmonicida*	Yes	
	S121	*A. salmonicida*	NZ_CP022175.1	*A. salmonicida*	Yes	
	A449	*A. salmonicida*	NZ_CP000644.1	*A. salmonicida*	Yes	
	A527	*A. salmonicida*	NZ_CP022550.1	*A. salmonicida*	Yes	
A. schubertii	CECT 4240 [T]	*A. schubertii*	NZ_CP013067.1	-	No	1/3 (33)
	LF1708	*A. schubertii*	NZ_CP039611.1	*A. schubertii*	No	
	WL1483	*A. schubertii*	NZ_CP013067.1	*A. schubertii*	Yes	
A. sobria	CECT 4245[T]	*A. sobria*	NZ_CDBW00000000.1	-	yes	2/2 (100)
	CU5	*Aeromonas sp.*	NZ_CP023817.1	*A. sobria*	Yes	

Species	Strain	Identified (NCBI)	Accesion number (genome)	Re-identification	HypA Presence	Rate of hypA presence (%)
A. taiwanensis	CECT 7403 [T]	*A. taiwanensis*	NZ_CDDD00000000.1	-	No	0/4 (0)
	186	*A. taiwanensis*	NZ_QORJ00000000.1	*A. taiwanensis*	No	
	198	*A. taiwanensis*	NZ_QORK00000000.1	*A. taiwanensis*	No	
	1713	*A. taiwanensis*	NZ_QORL00000000.1	*A. taiwanensis*	No	
A. veronii	CECT 4257 [T]	*A. veronii*	NC_015424.1	-	yes	18/18 (100)
	AER39	*A. veronii*	NZ_AGWT00000000.1	*A. veronii*	Yes	
	AMC34	*A. veronii*	NZ_AGWU00000000.1	*A. veronii*	Yes	
	AMC35	*A. veronii*	NZ_AGWW00000000.1	*A. veronii*	Yes	
	A134	*A. veronii*	NZ_RSFC00000000.1	*A. veronii*	Yes	
	Hm21	*A. veronii*	NZ_ATFB00000000.1	*A. veronii*	Yes	
	Aer 397	*A. veronii*	NZ_AGWV00000000.1	*A. veronii*	Yes	
	Hm22	*A. veronii*	NZ_NKWH00000000.1	*A. veronii*	Yes	
	TTU2014-	*A. veronii*	NZ_LKKB00000000.1	*A. veronii*	Yes	
	X11	*A. veronii*	NZ_CP024930.1	*A. veronii*	Yes	
	B565	*A. veronii*	NC_015424.1	*A. veronii*	Yes	
	TH0426	*A. veronii*	NZ_CP012504.1	*A. veronii*	Yes	
	AVNIH1	*A. veronii*	NZ_CP014774.1	*A. veronii*	Yes	
	CB51	*A. veronii*	NZ_CP015448.1	*A. veronii*	Yes	
	17ISAe	*A. veronii*	NZ_CP028133.1	*A. veronii*	Yes	
	X12	*A. veronii*	NZ_CP024933.1	*A. veronii*	Yes	
	Hm22	*A. veronii*	NZ_NKWH00000000.1	*A. veronii*	Yes	
	E20102	*A. veronii*	NZ_QIMH00000000.1	*A. veronii*	Yes	
A. trota	CECT 4255 [T]	*A. enteropelogenes* [b]	NZ_CDDE00000000.1	*A. trota*	Yes	4/4 (100)
	Aer371	*A. enteropelogenes* [b]	NZ_RQKA00000000.1	*A. trota*	Yes	
	LK14	*A. enteropelogenes* [b]	NZ_LDWG00000000.1	*A. trota*	Yes	
	CECT 4487	*A. enteropelogenes* [b]	NZ_CDCG00000000.1	*A. trota*		
A. encheleia	CECT 4342 [T]	*A. encheleia*	NZ_LR134376.1	-	Yes	2/2 (100)
	NCTC12917	*A. encheleia*	NZ_LR134376.1	*A. encheleia*	Yes	
A. aquatilis	CECT 8026 [T]	*A. aquatilis*	c	-	Yes	-
A. aquatica	CECT 8025 [T]	*A. aquatica*	NZ_JRGL00000000.1	-	Yes	-
A. enterica	CECT 8981 [T]	*A. enterica*	c	-	Yes	-
A. eucrenophila	CECT 4224 [T]	*A. eucrenophila*	NZ_CDDF00000000.1	-	Yes	-
A. intestinalis	CECT 8980 [T]	*A. intestinalis*	c	-	Yes	-
A. finlandensis	CECT 8028 [T]	*A. finlandensis*	NZ_JRGK00000000.1	-	Yes	-
A. lacus	CECT 8024 [T]	*A. lacus*	NZ_JRGM00000000.1	-	Yes	-
A. piscicola	CECT 7443 [T]	*A. piscicola*	NZ_CDBL00000000.1	-	Yes	-
A. popoffi	CECT 5176 [T]	*A. popoffi*		-	Yes	-
A. sanarelli	CECT 7402 [T]	*A. sanarelli*	NZ_CDBN00000000.1	-	Yes	-
A. tecta	CECT 7082 [T]	*A. tecta*	NZ_CDCA00000000.1	-	Yes	-
A. australiensis	CECT 8023 [T]	*A. australiensis*	NZ_CDDH00000000.1	-	No	-
A. cavernicola	CECT 7862 [T]	*A. cavernicola*	NZ_PGGC00000000.1	-	No	-
A. crassostreae	CECT 8982 [T]	*A. crassostreae*	c	-	No	-
A. diversa	CECT 4254 [T]	*A. diversa*	NZ_APVG00000000.1;	-	No	-
A. fluvialis	CECT 7401 [T]	*A. fluvialis*	NZ_CDBO00000000.1	-	No	-
A. molluscorum	CECT 5864 [T]	*A. molluscorum*	NZ_AQGQ00000000.1;	-	No	-
A. rivuli	CECT 7518 [T]	*A. rivuli*	NZ_CDBJ00000000.1	-	No	-
A. simiae	IBS S-6874 [T]	*A. simiae*	NZ_CDBY00000000.1	-	No	-

a Two different sequences with the same name.

b. *A. enteropelogenes* is a synonym of *A. trota.*

c. Draft genomes not freely available.

Tabla S2. A total of 95 true positives protein sequences of HypA from 60 genus and 77 species of bacteria obtained from Prosite and Uniprot databases.

Species	Protein ID	Species	Protein ID
Actinobacillus succinogenes	HYPA_ACTSZ (A6VPH7)	*Legionella pneumophila*	HYPA_LEGPH (Q5ZSP1)
Aeromonas hydrophila	HYPA_AERHH (A0KL76)	*Legionella pneumophila*	HYPA_LEGPA (Q5X252)
Aeromonas salmonicida	HYPA_AERS4 (A4SLV6)	*Legionella pneumophila*	HYPA_LEGPL (Q5WTX7)
Anabaena variabilis	HYPA_ANAVT (Q3M482)	*Legionella pneumophila*	HYPA_LEGPC (A5IEY2)
Aquifex aeolicus	HYPA_AQUAE (O67133)	*Methanococcus jannaschii*	HYPA_METJA (Q57667
Anacystis nidulans	HYPA_SYNP6 (P94160)	*Methanosarcina barkeri*	HYPA_METBF (Q46BF1)
Archaeoglobus fulgidus	HYPA_ARCFU (O28904)	*Methanosarcina acetivorans*	HYPA_METAC (Q8TRN7)
Azotobacter chroococcum	HYPA_AZOCH (Q43948)	*Methanosphaera stadtmanae*	HYPA_METST (Q2NEZ5)
Azoarcus sp.	HYPA_AZOSB (A1KC59)	*Methanothermobacter* [illegible]	HYPA_METTH (O26876)
Azotobacter vinelandii	HYPA_AZOVI (P31879)	*Methylacidiphilum infernorum*	HYPA_METI4 (B3DWG1
Beijerinckia indica	HYPA_BEII9 (B2IJ38)	*Methanobrevibacter smithii*	HYPA_METS3 (A5UJD5)
Bradyrhizobium diazoefficiens	HYPA2_BRADU (Q45256)	*Methanopyrus kandleri*	HYPA_METKA (Q8TV53)
Bradyrhizobium sp.	HYPA_BRASO (A4YNU5)	*Methanosarcina Frisia*	HYPA_METMA (Q8PUL7)
Campylobacter jejuni	HYPA_CAMJE (Q9PHP0)	*Nitratiruptor sp.*	HYPA_NITSB (A6Q3N0)
Chloroflexus aurantiacus	HYPA_CHLSY (B9LLZ7)	*Nostoc punctiforme*	HYPA_NOSP7 (Q9EYH3)
Chloroflexus aurantiacus	HYPA_CHLAA (A9WK98)	*Nostoc sp.*	HYPA_NOSS1 (O30461)
Chloroflexus aggregans	HYPA_CHLAD (B8G3M6)	*Paracoccus denitrificans*	HYPA_PARDP (A1B6P4)
Chlorobaculum tepidum	HYPA_CHLTE (Q8KBI8)	*Pectobacterium atrosepticum*	HYPA_PECAS (Q6D7U0)
Chlorobium limicola	HYPA_CHLL2 (B3EEL6)	*Pelodictyon phaeoclathratiforme*	HYPA_PELPB (B4SCQ5)
Chlorobium luteolum	HYPA_CHLL7 (Q3B2W3)	*Polaromonas naphthalenivorans*	HYPA_POLNA (A1VNP5)
Chlorobium vibrioforme	HYPA_CHLP8 (B3QQ33	*Pyrococcus furiosus*	HYPA_PYRFU (Q8U357)
Chlorobium phaeobacteroides	HYPA_CHLPB (B3EL96)	*Pyrococcus horikoshii*	HYPA_PYRHO (O59267)
Cupriavidus necator	HYPA_CUPNH (P31901)	*Prosthecochloris aestuarii*	HYPA_PROA2 (B4S9J3)
Dechloromonas aromatica	HYPA_DECAR (Q478M5)	*Psychromonas ingrahamii*	HYPA_PSYIN (A1SU80)
Dehalococcoides ethenogenes	HYPA_DEHM1 (Q3Z6L1)	*Pyrococcus abyssi*	HYPA_PYRAB (Q9V148)
Dehalococcoides mccartyi	HYPA_DEHMC (Q3ZYW6)	*Rhizobium leguminosarum*	HYPA_RHILV (P28154)
Dehalococcoides mccartyi	HYPA_DEHMB (A5FPQ5)	*Rhodobacter capsulatus*	HYPA_RHOCA (P26409)
Deinococcus radiodurans	HYPA_DEIRA (Q9RYJ6	*Rhodoferax ferrireducens*	HYPA_RHOFT (Q21R10)
Desulfococcus oleovorans	HYPA_DESOH (A8ZZG8)	*Rhodopseudomonas palustris*	HYPA_RHOP5 (Q07S91)
Desulfatibacillum aliphaticivorans	HYPA_DESAL (B8FH08)	*Rhodobacter sphaeroides*	HYPA_RHOSK (B9KKQ0)
Desulfovibrio magneticus	HYPA_DESMR (C4XS28	*Rhodobacter sphaeroides*	HYPA_RHOS1 (A3PLP3)
Desulfovibrio vulgaris	HYPA1_DESVH (Q729M2	*Rhodobacter sphaeroides*	HYPA_RHOS4 (Q3J0K8)
Desulfovibrio vulgaris	HYPA2_DESVH (Q729Q7)	*Rhodobacter sphaeroides*	HYPA_RHOSH (Q9REM0)
Esherichia coli	HYPA_ECOL6 (Q8FEL9)	*Rippkaea orientalis*	HYPA_RIPO1 (B7JUE3)
Esherichia coli	HYPA_ECOLI (P0A700)	*Salmonella typhi*	HYPA_SALTI (P64420)
Escherichia coli	HYPA_ECO57 (P0A701)	*Salmonella typhimurium*	HYPA_SALTY (P64419)
Geobacter daltonii	HYPA_GEODF (B9M4A8)	*Shewanella báltica*	HYPA_SHEB8 (A6WMK8)
Geobacter metallireducens	HYPA_GEOMG (Q39QV5)	*Shigella flexneri*	HYPA_SHIFL (P0A702)
Geobacter sulfurreducens	HYPA_GEOSL (Q74G73)	*Syntrophus aciditrophicus*	HYPA_SYNAS (Q2LVX4)
Geobacter sp.	HYPA_GEOSM (C6E310),	*Synechococcus elongatus*	HYPA_SYNE7 (Q31K36)
Geobacter uraniireducens	HYPA_GEOUR (A5GFC7)	*Synechococcus sp.*	HYPA_SYNP2 (Q8KX22)
Helicobacter pylori	HYPA_HELPJ (P0A0U5)	*Synechocystis sp*	HYPA1_SYNY3 (P74263)
Helicobacter pylori	HYPA_HELPY (P0A0U4)	*Synechocystis sp.*	HYPA2_SYNY3 (P73269)
Ignicoccus hospitalis	HYPA_IGNH4 (A8AAQ5	*Thermococcus gammatolerans*	HYPA_THEGJ (C5A3C3)
Klebsiella pneuomoniae	HYPA_KLEP3 (B5XV79)	*Thermococcus kodakaraensis*	HYPA_THEKO (Q5JIH3)
Klebsiella pneuomoniae	HYPA_KLEP7 (A6TCZ3	*Thermococcus onnurineus*	HYPA_THEON (B6YT59)
Lawsonia intracellularis	HYPA_LAWIP (Q1MRS4)	*Thermomicrobium roseum*	HYPA_THERP (B9L1X1)
		Trichodesmium erythraeum	HYPA_TRIEI (Q117V9)
